# Can support workers from AgeUK deliver an intervention to support older people with anxiety and depression? A qualitative evaluation

**DOI:** 10.1186/s12875-019-0903-1

**Published:** 2019-01-19

**Authors:** Tom Kingstone, Bernadette Bartlam, Heather Burroughs, Peter Bullock, Karina Lovell, Mo Ray, Peter Bower, Waquas Waheed, Simon Gilbody, Elaine Nicholls, Carolyn A. Chew-Graham

**Affiliations:** 1 Research Institute, Primary Care and Health Sciences, Keele University, Staffordshire, UK; 2 Midlands Partnership HS Foundation Trust, St George’s Hospital, Stafford, Staffordshire UK; 3 Family Medicine and Primary Care, Lee Kong Chian School of Medicine, Nanyang Technological University, Singapore, Singapore; 4 Chief Executive, North Staffordshire AgeUK, Staffordshire, UK; 5 Division of Nursing, Midwifery and Social Work, University of Manchester, Manchester, UK; 6 School of Health and Social Care, University of Lincoln, Lincoln, UK; 7 NIHR School for Primary Care Research, Centre for Primary Care, Division of Population of Health, Health Services Research and Primary Care, Manchester Academic Health Science Centre, University of Manchester, Manchester, UK; 8 Mental Health and Addictions Research Group, University of York, and Centre for Health and Population Sciences, Hull/York Medical School, York, UK; 9 Collaboration for Leadership in Applied Health Research and Care, West Midlands, UK

**Keywords:** Anxiety, Depression, Older adults, General practice, Primary care, Third sector services, Behavioural activation

## Abstract

**Background:**

Anxiety and depression often co-exist. These disorders are under-diagnosed and under-treated, specifically among older people, and lead to increased use of health and social care services and raised mortality. Older people report a reluctance to present to their GP with depression or anxiety symptoms due to perceived stigma about mental health problems, lack of acceptable treatments and the prioritising of physical health problems. Third sector organisations, who work closely with older people in the community, are well-placed to provide additional support. We developed a brief intervention based on principles of Behavioural Activation, with encouragement to participate in a group activity, for delivery by Support Workers from AgeUK. The aim of the study was to examine whether this brief intervention could be delivered to older people with anxiety and/or depression, with sufficient fidelity, and whether this approach was acceptable to patients, GPs and AgeUK Support Workers.

**Methods:**

Semi-structured interviews with older people with self-reported anxiety and/or depression (who received the intervention), Support Workers and GPs to assess acceptability of the intervention and impact on routine care. A constant comparative approach was used to analyse the data. Intervention sessions between Support Workers and older people were digitally recorded and reviewed by the research team to assess fidelity.

**Results:**

The Support Workers delivered the intervention with fidelity; access to the training maual and ongoing supervision were important. Older people found the intervention acceptable and valued the one-to-one support they received; group activities suggested by Support Workers were not valued by all. GPs recognised the need for additional support for vulnerable older people, but acknowledged they could not provide this support. Participation in the study did not impact on GP routine care, other than responding to the calls from the study team about risk of self-harm.

**Conclusions:**

Support Workers within AgeUK, can be recruited and trained to deliver an intervention, based on the principles of Behavioural Activation, to older people with anxiety and/or depression. The training and supervision model used in the study was acceptable to Support Workers, and the intervention was acceptable to older people and GPs. This model has the potential to contribute to improving the support and care of older people in primary care with anxiety and depression. Further testing is required in a full trial.

**Trial registration:**

Trial registration number ISRCTN16318986.

Registered 10/11/2016.

**Electronic supplementary material:**

The online version of this article (10.1186/s12875-019-0903-1) contains supplementary material, which is available to authorized users.

## Background

Depression is a major global public health burden; by 2030 depressive disorders are predicted to be the second leading cause of disease burden and disability worldwide [1]. Untreated anxiety and depression leads to increased use of health and social care services, and raised mortality [2]. Anxiety and depression occur frequently across all age groups and often co-exist, prevalence of depression is reported between 10 and 20% amongst older people [3, 4]. Demographic changes mean that even if prevalence rates were to remain stable, the growing numbers of older people will lead to large increases in the demand for treatment for these disorders in this population [5].

Depression and anxiety are more prevalent in people with long-term physical condition(s) and more than seven times more common in those with two or more chronic physical conditions [6]. Thus, mental and physical health problems tend to become entwined and manifest in complex co-morbidity, worsening prognosis and adversely affect overall quality of life [6–8]. As co-morbidities are common in later life (36% of people aged 65–74 and 47% of those aged 75 and over have a limiting chronic illness) they constitute a serious risk factor for developing depression and/or anxiety in this population [9].

Moreover, depression and loneliness are strongly associated; longitudinal research has reported loneliness as an independent risk factor for future depression [10, 11]. Loneliness is associated with a high degree of morbidity including poor physical and mental health/function, increased health and social service utilization, higher use of medication use, early entry into residential or nursing care, and above all increased mortality [12–17]. Loneliness is often a consequence of bereavement, particularly in spousal bereavement or divorce, and with low social interaction is predictive of suicide in older age [18].

Anxiety and depression remain poorly detected and treated in primary care [19]. For older people with undetected depression, longer-term prognosis is poorer than for those with depression whose General Practitioner (GP) is aware [20]. One impediment to detection is that older people may not present to their GP because of the stigma they perceive about mental health problems [21]. Older people with LTCs may normalize their depression, or view their long-term physical condition(s) as a ‘justifiable’ cause of low mood [22–24]. As a result, older people may hold negative views about help-seeking [25]. Diagnosis and treatment led by a narrow bio-medical model may overlook important social and contextual factors of mental health, which can inform management [24]. One way around this is to treat people with mild to moderate depression and anxiety in a way that under-served individuals, such as older people, find non-stigmatising.

The NICE guidelines for depression [21] and anxiety [26] advocate a stepped care management approach with those who have mild to moderate anxiety and depression being offered advice about lifestyle by GPs as Step 1, and low intensity interventions which may include provision by non-statutory or third sector bodies as Step 2. There is limited evidence of the effectiveness of such providers in improving patient outcomes. NICE Guideline 123 [27] emphasise the need to promote access to services for people with common mental health disorders for a range of socially excluded groups including older people, with interventions in the person’s home, and/or assistance with travel, and sign-posting (i.e. referring) to self-help and support groups.

In terms of current treatments, anti-depressants may not be an acceptable option for older people, concordance may be poor [27], and evidence repeatedly suggests that older people are not referred for ‘talking treatments’ [28]. There is evidence that befriending (a one-to-one intervention) is effective in reducing depression in older people [29]. Lester et al. [30] suggest that befriending provides older people with opportunities to develop social ties that they perceive as reciprocal, to share intimacies and establish trust. However, according to a systematic review of health promotion interventions for socially isolated and lonely older people one-to-one interventions for older people are insufficient. The review found that nine of the ten effective intervention studies included were group activities with an educational or support input, whereas six of the eight ineffective intervention studies provided one-to-one social support, advice and information, or health-needs assessment [31]. Group-based activities that focus on a shared interest are preferred by older people to one-to-one support or general social groups; however, groups advertised for ‘lonely older people’ are not considered desirable or helpful [32]. Thus, befriending alone is unlikely to achieve lasting effect, and the practitioner delivering the intervention needs to consider social context and social support [33].

Behavioural Activation (BA) is a short-term cognitive-behavioural therapy (CBT) based intervention, known to be effective in the management of depression and which can be delivered by non-mental health trained practitioners [34, 35]. BA focuses on activity scheduling to encourage participants to approach activities that they may have previously enjoyed but are currently avoiding, or to develop new activities that take into account increasing life changes (for example, loss of spouse), and consider the function of cognitive processes (e.g. rumination) that serve as a form of avoidance. Participants are thus supported to refocus on their goals and valued directions in life. Behavioural therapies have been shown to be effective in older people [36, 37].

Previous studies have explored ways to improve access to mental health care for marginalised groups [38]. In the Improving Access to Mental Health in Primary Care (AMP) Programme [39] psychological well-being practitioners (seconded from local Improving Access to Psychological Therapies (IAPT) services) delivered a brief psychosocial intervention to older people, who found this intervention acceptable [40]. Whether third sector workers can deliver such an intervention to older people, and whether it is acceptable to patients and effective in improving outcomes, is unknown but could be cost effective.

NOTEPAD was a pilot study to determine if it is feasible to recruit and randomise patients, to pilot procedures, and to conduct a process evaluation in order to provide essential information and data to inform a proposal for a full randomised trial [41, 42]. Here, we report the process evaluation, the aim of which was to explore whether AgeUK SWs could deliver the NOTEPAD psychosocial intervention to older people, with sufficient fidelity; and whether this approach was acceptable to patients, general practitioners and the third sector providers.

### The NOTEPAD feasibility study

Full details of the study are reported elsewhere [42]. In brief, patients were recruited through six primary care practices in North Staffordshire. Practice lists from the participating general practices were searched for patients aged over 65 years of age. GPs screened the resulting lists to identify those who met the inclusion criteria. Patients scoring 10 or higher on either the PHQ9 [43] [Kroenke et al., 2001] or the GAD7 [44] [Lowe et al., 2008], which had been posted out following screening of GP lists, and who then consented to further contact formed the sample for invitation into the feasibility study and were randomly allocated to usual care or the intervention. All consenting patients received a research nurse (RN) visit at baseline and at four-months. At both visits the PHQ9 and the GAD7 were repeated.

#### Usual care arm

Participants randomised to the usual care arm received whatever care was judged to be indicated by the primary care practitioners in contact with them. No constraints were placed on what constituted ‘usual care’.

#### Intervention arm

Participants randomised to the intervention arm were contacted by the AgeUK Support Workers (SWs) and offered an individual appointment either in the participant’s home or at a local third sector service (depending on participant preference). It was anticipated there would be between 4 and 6 contacts between the participant and the SW, in a combination of face to face and telephone contact within a four-month period from baseline. The intervention was intended to be tailored to participant preferences, so there was flexibility regarding the precise number of sessions, interval, mode of delivery and format. The identification of group activities was led by participant interests with support provided to access these opportunities. Intervention group participants also received treatment as usual from their general practice.

## Methods

Two methods were used in the evaluation: a) fidelity checking on delivery of the intervention; b) semi-structured individual interviews with older people and SWs. Interviews or focus groups (according to preference) with GPs in participating practices.

### Recruitment to the process evaluation

#### Fidelity checking

The content of the intervention was monitored for fidelity by digitally recording the first two sessions the SW has with each participant. These digital recordings were checked against a fidelity checklist (Additional file 1), by CC-G and HB, to assess whether components of the SW sessions intended to be included, and focused on during training, were demonstrated by the SW in the recorded session.

### Interviews

#### Older people

To assess acceptability of the intervention, those randomised to the intervention arm were invited – at their four-month visit from the RN – to take part in a semi-structured interview. We also sought to conduct interviews with any older person who dropped out of the intervention.

A letter was sent to participants after their follow-up appointment with the RN to advise them that a researcher would contact them by telephone and arrange a time and date for interview. Consent for this contact was obtained at entry into the study. Sampling was guided by baseline characteristics to ensure views from a diverse sample were gathered. The participants who took part in the process evaluation interviews were offered a £20 shopping voucher as a ‘thank-you’ for participating.

#### General practitioners

Letters were sent to GPs in participating practices, inviting them to participate in a semi-structured interview.

#### Support workers

SWs were employed by AgeUK... Letters were sent to the SWs, followed up with e-mail and telephone contact, to arrange a time for an interview. The SWs agreed to participate in the interview as part of their appointment to the SW role. They were invited for interview within four weeks of their last appointment with their last participant.

#### Data generation

Interviews with study participants were conducted by BB or HB, at a time and place convenient to the participants. General Practitioners were offered the option of a telephone interview, or joint interviews with colleagues in the practice. Topic guides were developed for each participant type: patient participants (in the intervention arm) (Additional file 2), patient participant (dropouts) (Additional file 3), GPs (Additional file 4), and SWs (Additional file 5).

### Data analysis

#### Analysis of digitally recorded consultations (fidelity checking)

A descriptive analysis was produced. Data collected from the audio recordings will be available for future thematic analysis.

#### Analysis of interview data

The interviews were transcribed verbatim, the transcripts formed the data for analysis. Data were stored, managed and analysed using NVivo software. Initially the data were analysed using the constant comparison method [45], within each data-set (patient participants, SWs and GPs). Analysis was then conducted across the three data-sets presented using the principles of Framework Analysis [46] – a method that is appropriate for applied policy research and allows the development of an understanding of how the intervention was implemented (or not) and operationalised by respondents. A team of researchers (CCG, BB and HB) conducted analysis individually, and then agreed themes through discussion. Conducting analysis with researchers of different professional backgrounds increases the trustworthiness of the analysis [45].

## Results

### Fidelity

Tables 1, 2 and 3 show the results of fidelity checking the recorded sessions. 50 sessions were delivered in total by 4 SWs. 22 sessions were digitally recorded; 14 sessions of which were first sessions, 6 were second sessions and 2 were subsequent sessions (recorded by the SW when the client gave permission for recording later in the intervention).

**Table 1:** NOTEPAD Fidelity Checklist - First sessions

	YES	Partially	NO
Verbal explanation given of the NOTEPAD study	13	1	
Explanation of the evidence for the beneficial effects of social participation and depression	9	3	2
Evidence of exploring the older person’s problems	13	1	
Assessment of risk	8	3	3
Activities/social participation goals discussed	14		
Activity/social participation goals set	12	1	1
The NOTEPAD personal file given along with a verbal explanation of how to use it	10	4	
Signposting – (e.g. exercise groups, craft classes etc.)	9	4	1
Participant understanding of what has been discussed and agreed is checked	11	2	1
Barriers/motivators to increasing activity discussed and/or addressed	11	2	1
Next session discussed and arranged (face to face or telephone)	13		1

**Table 2:** NOTEPAD Fidelity Checklist – Second sessions

	YES	Partially	NO
Review mood - mood thermometers	3	1	2
Review progress - diary	4	1	1
Feedback given regarding any progress made	6		
Barriers/motivators to increased activity/participation discussed and/or addressed	4	2	
Activity/social participation goals discussed	3	1	2
Activity/social participation goals set	2	2	2
Signposting – (e.g. craft groups, adult learner classes etc.)	2	1	3
Remind about use of NOTEPAD personal file	2	1	3
Relapse prevention / staying well strategies discussed (e.g. support and guidance)	2	3	1
Possible personal issues/difficulties encountered whilst increasing activity/social participation.	3	3	
Relevant contact details are given in case of any problems, issues or further advice required	4		2

**Table 3:** NOTEPAD Fidelity Checklist – Subsequent sessions

	YES	Partially	NO
Review mood – mood thermometers	1		1
Review progress (diary)	2		
Feedback given regarding any progress made	2		
Barriers/motivators to increased activity/participation discussed and/or addressed	2		
Activity/social participation goals discussed	2		
Activity/social participation goals set	2		
Signposting – (e.g. craft groups, adult learner classes etc.)	1	1	
Remind about use of NOTEPAD personal file	1	1	
Relapse prevention/staying well strategies discussed (e.g. support and guidance)	2		
Possible personal issues/difficulties encountered whilst increasing activity/social participation.	2		
Relevant contact details are given in case of any problems, issues or further advice required	2		

Reasonable delivery of the intervention was achieved, particularly in the first session.

### Semi-structured interviews

#### Study participants

We outline the key findings from the interviews with 17 patient participants. Of the 18 who were allocated to the intervention group, two people did not complete the intervention. These are denoted as ‘dropouts’ and were both interviewed. We interviewed SWs (*n* = 6) and GPs (*n* = 12).

The mean duration of interviews was 23 min for patients (range 12 to 68 min), 37 min for SWs (range 30 to 51 min) and 23 min for the GPs (range 11 to 28 min).

Tables 4, 5 and 6 give details of the participants.

**Table 4:** Baseline characteristics of the interview participants

Study ID	Gender	Ethnic group	Employment status	Marital Status	Living situation	General Health	Taking Medication for low mood or stress	Longstanding illness, disability	Completer/Dropout
11	Male	White British	Retired	Married	Live with more than one other person	Poor	No	Yes	Completer
145	Male	White British	Retired	Divorced or separated	Live with another person	Poor	Yes	Yes	Completer
441	Male	White British	Carer	Married	Live with another person	Fair	No	Yes	Completer
467	Male	White British	Missing	Married	Live with another person	Fair	No	Yes	Completer
589	Female	White British	Retired	Divorced or separated	Live alone	Poor	No	Yes	Completer
1061	Female	White British	Retired	Widowed	Live alone	Poor	No	Yes	Dropout
1093	Female	White British	Retired	Married	Live with more than one other person	Poor	Yes	Yes	Completer
2427	Female	White British	Retired	Widowed	Live alone	Very good	Yes	Yes	Completer
2589	Female	White British	Retired	Widowed	Live alone	Fair	No	Yes	Completer
2662	Female	White British	Missing	Widowed	Live with another person	Poor	No	Yes	Completer
2777	Male	White British	Retired	Married	Live with another person	Poor	Yes	Yes	Completer
2945	Female	White British	Retired	Married	Live with another person	Excellent	Yes	No	Completer
2977	Male	White British	Retired	Married	Live with another person	Good	Yes	Yes	Completer
3009	Female	White British	Retired	Co-habiting (living as married)	Live with another person	Poor	Yes	Yes	Completer
3060	Male	White British	Missing	Married	Live with another person	Very good	No	Yes	Dropout
3512	Male	White British	Retired	Married	Live with more than one other person	Very good	No	No	Completer
3560	Female	White British	Retired	Widowed	Live alone	Good	Yes	Yes	Completer

Patient participants (*n* = 17); mean age = 74.1 yrs.; range = 66-85 yrs

**Table 5:** GP Interviewees

General Practice	List size	Interviewees	Gender (M or F)
Practice 1	4648	2	M
Practice 2	7028	3	2 M, 1F
Practice 3	7255	1	M
Practice 4	10,978	4	2 M, 2F
Practice 5	5545	2	2 M

Note, some interviews with GPs were conducted in groups, at the end of practice meetings. Some one-to-one interviews were conducted over the telephone

**Table 6:** SW Interviewees

SWs	*N* = 6
Female	5
Age	45–50 years (2)
	55–60 years (2)
	60–65 years (1)
	65–70 years (1)

Limited data is given about the SWs, to ensure anonymity is preserved. All interviews were conducted face to face

All interviews were conducted face to face in patients’ homes.

### Perspectives of patient participants

A number of key, inter-linked themes emerged from analysis of the patient participant interview data: recognising depression and the long-standing nature of mental health problems, co-morbid physical and mental health problems, loneliness, support received, and views and reflections on the NOTEPAD intervention.

#### Recognising depression and the long-standing nature of mental health problems

Participants recognised that they were suffering from depression, and that it was currently, and had previously, caused difficulties in their lives:

*“I’ve been up and down, I mean I’ve had it 50 years to tell you the truth so I had it in my 20s yeah. …the first time I was depressed in [my] early 20s.”* P3512.

Linked to the longstanding nature of their problems, people reported diverse experiences of treatments ranging from interventions by GPs and counselling services through to psychologists and psychiatrists, and pharmaceutical approaches:

*P: I’ve been treated for depression since – when was it – 1998. I’m still on tablets now for depression. I’ve been to see, talk to somebody, like a psychiatrist... It weren’t a psychiatrist, it was…. [trails off].*

I: A psychologist?

*P: Something like that. P3060 [dropout].*

#### Multiple and complex problems

Older people reported multiple illnesses:

*“Yes, yes, I’ve got it all. You name it, if it’s free I have it. Now I’ve got, what is it? Cholesterol, COPD, diabetes two, blood pressure. I’ve just been up and had blood taken this morning. Mmm, I’ve got ‘em all.” P11.*

In addition to physical health problems, some participants’ experiences were compounded by difficulties with their families:

*“There are currently, and there has been for about 18 years, quite severe family problems and they’re getting worse as well with certain, certain parts of the family. And that’s causing a lot of stress and distress.”* P589.

Other participants reported caring responsibilities:

*“I was at a stage where I was running backwards and forwards to my mother and then when my mother died I suppose it made it easier because then it was only my husband but I mean he got, I had to take him the toilet, in other words it was full on. I had to wash him, dress him, everything.”* P3650.

Some participants reported financial difficulties which they felt impacted on their health:

*“The disability living allowance, it’s, it’s gone to PIP now and I had my assessment …but I’m just waiting to hear from that now, to, you know, know whether I’m going to keep me car and that. It’s just – because I’d be lost without the car because …..I can’t walk and get buses now, I can’t even sit on a bus, so I’m worried sick about that because I don’t know what I’d do if I – do you know what I mean?”* P145.

#### Loneliness

Some study participants expressed feelings of loneliness and isolation, acknowledging that loneliness exacerbated their feelings of anxiety and depression:

*“I do suffer from anxiety and depression and I worry a lot. I worry tremendously. I never used to worry this much but I do now and I think it’s because… I think it’s because I’m on my own… every little thing… and when I draw the curtains at night I’m thinking, ‘oh gosh what’s that and it’s probably absolutely nothing. Just a car passing’.”* P2427.

Respondents who admitted to feeling lonely described their families as living far away or busy with work or their own family commitments:

*“I’ve got two sons who try very hard to be supportive but are quite some distance away, work seven days a week, have family of their own, so are not as available.” P589.*

#### Support received

GPs were seen as an important source of support for the variety and complexity of problems, particularly physical, described. However, a number of patients reported that the treatment offered by their GP for low mood had been ineffective in the past, particularly where a pharmaceutical approach was taken, as P1093 describes:

I: Okay have you spoken to your GP about it or…?

*P: I have done, yeah but they just give you tablets.*

I: Right so have you taken tablets?

*P: Yeah, I do, yeah.*

I: You do? For your mood?

*P: Yeah, yeah.*

I: Yeah, and do you find that helps or not?

*P: Not really, no.*

Moreover, patients had little expectation that their GP could offer any alternative due to time constraints on the length of consultations:

*“I know, having worked with GPs, you know, their time is so short it’s very difficult to deal with any problems but particularly mood and depression. It’s very hard for them to deal in the surgery with how people are feeling because, you know, it’s not a five- or ten-minute thing, is it?” P2977.*

#### Experiencing the intervention

All the participants who completed the intervention suggested that it was acceptable, even though some were initially uncertain about what it might involve:

*“At first I wasn’t quite sure what it was about, I couldn’t quite grasp what it was about …. and then as time went on a bit I started to realise you were really trying to find out what elderly people want and what their needs are really. And then it sort of got a bit more interesting to me and I thought, ‘oh somebody actually asking me [what you want]… I just felt that my answers mattered because I feel as if I’m just one in millions as just feel the same.”* P2427.

The participants reported that the empathic approach of the SWs and the time available to be listened to by the SW were valuable experiences, regardless of whether they considered themselves lonely or not. The personal qualities of the SW were particularly commented upon:

*“She actually listened which a lot of people don’t do….. she very quickly seemed to grasp the struggle I was having”* P2589.

*“I thought she was quite professional; she was empathetic and I felt she was listening to me.” P3009.*

Participants also reported that the practical support offered by the SWs was helpful and reported receiving help with claiming benefits and filling in forms, in addition to signposting to local groups and activities:

*“She gave me a lot of contact information about organisations that could help with bereavement, for instance and benefits, finance things and she also found me a support group called [*name of local group*]”.* P589.

*“And he found out other interests or other things I could do in [the] area….he found indoor bowling as well, down the leisure centre. Then I go swimming occasionally as well. It’s different groups I didn’t know about in [the area], community groups where you can go. Just like to play dominoes or a book club or things like that.”* P2977.

In helping older people identify their goals, respondents suggested that the SWs also went beyond simply mentioning possible activities:

*“And she said, ‘Do you want me to come with you?’ And I said, ‘Yes, that would be nice.’ So she came with me the first time. She didn’t stay for the whole session but she said, before we went in, she said; ‘Let me know if you feel at any time that you’re okay and that you don’t need me there,’ sort of thing. And so she went halfway through. She went by our agreement.”* P589.

Some patients reported attending groups to be helpful:

*“It’s quite a positive thing. Everybody sits around and talks about what sort of day or week they’ve had and things come out, you know? Like I was struggling about my dad, and about some other family issues, and the rest of the group all sort of say, you know, they sort of give you encouragement. Like saying, you know, ‘Well, you’ve done really well to do this and do that‘, So it was good and I think I will go again.”* P3009.

Other people reported that they did not want to attend social or activity groups, even when they acknowledged that the SWs seemed to have tried hard to find a group to match the person’s interests, and help to overcome psychological and/or practical barriers to attending. Those people who did not find attending social or activity groups appealing did not feel themselves to be lonely or in need of company or activity. They lived with spouses or other family members or were busy with caring responsibilities:

*“She [the SW] desperately wanted me to go to erm places that I haven’t been to before like there’s a leisure place in [local town] where they do various clubs. I didn’t want to go. I really didn’t want to meet people I didn’t know.” P1061 [dropout].*

“*But the more I thought of it I didn’t really want that ‘cause I aren’t on me own, I’ve got me family and me wife here. I thought it was more for people on their own sort of thing, you know, no company or anything like that. So I was quite happy myself.”* P3060 [dropout].

Participants reported that they valued activity that they felt to be personally meaningful and were not interested in groups that did not contribute in that way:

*“Yes, well it didn’t help but he* [the SW] *was helpful. Helpful in the list of things. He did try to get me to go to Men in Sheds and all that. I just didn’t fancy driving all that way to sit and drink tea. But I don’t drink tea and so I just couldn’t see a future for me there of any kind.”* P11.

However, those who engaged with the intervention, but did not want to attend groups, still reported the time spent with the SW, being listened to, was beneficial to them:

“*You see, I don’t want to go out to these groups as I’ve explained cos I can’t…..but having somebody coming to talk to you relaxes you and all that, it’s great.”* P1093.

Most participants reported that they felt better having met with the SW, as participant 441 below who was main carer for his wife reported, attributing this change to the SW visiting:

*“We’re working better now than it has done in the past. She [the SW] has done wonders. I’ve got [my wife] used to the routine and I’m on a routine which we can cope with, I can cope with well. And I think if anything [my wife’s]‘s better than she has been [right] she’s yeah, she is, she’s better.”* P441.

**Fig. 1:**
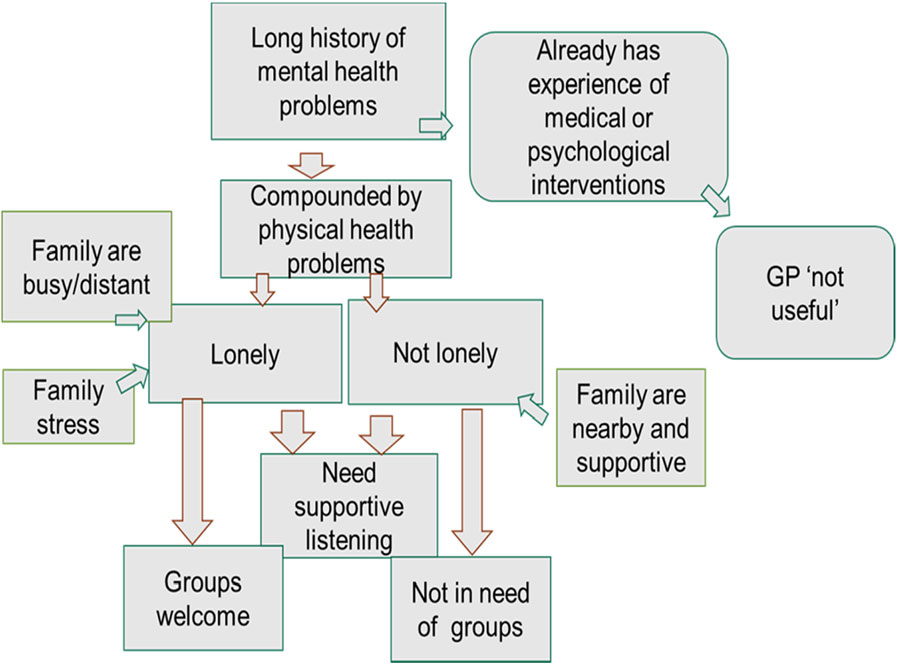
Conceptual model

The following model (Fig. 1) illustrates how the distinction between loneliness and depression is an important one. Those who described themselves as being lonely valued supportive listening as well as help to access meaningful group activity. The group who characterised themselves as ‘not lonely’ because they have support from family nearby, did not want to attend groups but still wanted and valued being listened to. This model was checked out with the study PPIE group. We debated the code ‘GP not useful’, and considered alternative ways of expressing this, but the group felt that this phrase represented the data from both study participant and GP data-sets.

### Perspectives of support workers

The interviews with the SWs focused on the training, supervision, delivering the intervention, and suggestions for refining the intervention. Data are provided to support each of the themes presented, with participant identifiers.

#### Engagement with the study

The SWs all reported that they found the idea of the study novel and interesting:

*“I was looking for, you know, a bit of a new challenge really. Because my son’s gone off to university and, and I just thought ‘Oh, do something a bit different’, that I’m interested in, you know. ‘Because I’m quite interested in helping people, really. I’ve always been in the caring profession really, so. It just sounded interesting, really.”* SW1.

*“I mean overall, I think it was – it’s been really good to be involved. I thought kind of the initial information we got about what and why, you know, I thought was really well sort of researched and it seemed like it was a really good idea.”* SW3.

#### Prior experience

The SWs described a wealth of prior professional experience and some had completed courses in counselling:

*“I’d been a Teacher in a former life and then became a trainer in the voluntary sector….I did some study, it was just a year’s psychology for everyday life and I’d done psychology for my degree years ago anyway.”* SW 6.

*“I did counselling courses when I was young, maybe about 10 years ago now so I have a bit of a basic foundation of one-to-one interaction with people.”* SW4.

Thus, the SWs recruited from AgeUK had broad knowledge and skills on which the NOTEPAD training could build on.

#### Experiences of training

The SWs all spoke of experiencing anxiety prior to and during the training:

*“We were all scared, we were all really scared. I mean, we were very well supported on the training and, to be kind of, doing the skills practice and have people sitting there observing, was initially very scary.”* SW6.

*“I mean overall, I think it was – it’s been really good to be involved. I thought kind of the initial information we got about what and why, you know, I thought was really well sort of researched and it seemed like it was a really good idea [right]. I think – yeah, the training was really good.”* SW3.

All of the SWs reported that the training manual was essential:

*“It, it was very helpful. You know, it rejogs your memory, builds on the foundations that you had in, yeah. It refreshes you, and I found that I was reading, the session one every, time I was going into the person and reading session two every time I was going in to see them, see them the second time and the third time and the familiarising myself with the mood thermometers, and explaining that to them.”* SW4.

Some of the SWs described how they had made their own summaries of the key messages in the manual, which were vital in the first couple of encounters, but eventually needed less as they became more confident working with their clients:

*“I made my own prompt sheet because you feel a bit, like everything’s entering your head at once sort of thing. So, it was just sort of all the things. That was one of the most difficult things. There were all sorts of aspects that you had to include but, one of the tricky things was how do I order this. Should I say, should I broach that part before that? Do I make the list before we go into this part? So, the prompt sheet was really useful for that.”* SW5.

As the SWs became more experienced, the manual was used less:

*“I did read, more or less read through that each time, you know, well I wouldn’t say before every contact, because the further I went on with contact and settled into it and could actually see, you know, see BA in practice and how actually, you were able to engage with somebody and it did, it did make sense, that gave me a bit more confidence and I felt I was internalising it, I wasn’t so focussed on, oh you know, I’ve got all these things written down, how am I going to remember it all, because it was, it was making sense.”* SW6.

#### Delivering the intervention

Most of the SWs reported experiencing anxiety following the training and prior to visiting their first client and carrying out the intervention:

*“I felt completely overwhelmed by what I was hearing, I think it was far, far more [complex] than I had ever anticipated….because I was so nervous, throughout the first 10 minutes, my heart, you know, I could feel my heart was going like this, I could barely speak, because I was stuttering and I thought for God’s sake, get a grip, get a grip of yourself.….. I felt this pressure…. I felt quite a responsibility that, you know, we’d had this time invested, this knowledge given to us and I didn’t want to let anybody down.”* SW6.

SWs reported that there were particular areas that they initially found difficult, such as doing the risk assessment, but which they became more confident in as they saw more clients:

*“I’m a lot more comfortable now after seeing six people than when I started. It’s just a shame he was the very first one you see? It was like baptism of fire really. But I managed to, broach the subject with everybody, and I was getting a lot better at it. … So, yeah, I’m much more comfortable than I was at the start. But I found the training very useful.”* SW 5.

#### Utilising existing knowledge

As they were already employed by AgeUK and were running activity groups, SWs felt they had a comprehensive knowledge about benefits and resources, services and support groups available in the local area:

*“Obviously I know about the AgeUK and benefit type things and I use the internet a lot to find social groups and things that were available. And perhaps my local knowledge, because a lot of the, clients I got, because I think they were from [*names Health Centre]*, the last group. Because I run my befriending scheme in* [names town] *I knew what was available there so that was useful.”* SW5.

Recruiting SWs with this local knowledge to the study was key.

#### Dealing with complexity

The SWs found dealing with the complex needs of some clients challenging and requiring careful thought beyond simply delivering an intervention:

*“I’m thinking, how am I going to help this man, how am I going to help him? But if I’d said ‘oh well, you know, why don’t you ring [a group]’, that would have been completely inappropriate. ……. And just thinking, you know, what, how, you know? I can’t wave a magic wand and make this go away, but it wasn’t about that, and then, you know, you come away and you make your notes and you reflect.”* SW6.

The SWs sometimes found that organised groups, which they had prior knowledge of, may not be appropriate for their clients, and finding alternatives required careful investigation, with often a number of suggestions offered to clients:

*“I’d kind of gently kept coming back to these groups and I thought, ‘Maybe this isn’t kind of what they want’. So I asked them, ‘Well,’ you know, ‘kind of what, what – where do you go? What, what do you do? What, what do you like doing?’ and they said, ‘Going to garden centres. He loves B&Q. Doing things around the house’ and I said, ‘Right, okay. It doesn’t have to be a Bladder Cancer Support group,’ you know. ‘If, if that’s what you would like to go to...’ you know, ‘what about planning it?’”* SW3.

#### Achieving success

Reflecting on the challenges in working with clients with complex needs, SWs reported a sense of satisfaction when goals were achieved:

*“She did [go to the group], she did! Oh it was, I kind of ran round the office going, ‘yes, yes’. It was great. I was just in the office with my colleague who runs these groups and, my colleague took a phone call and then my colleague said to me the lady’s name and she said, ‘well, she’s just phoned up to enquire about the computer groups and she’s going to come along on Thursday’. So that was thrilling. That was really, really thrilling…”* SW6.

SWs also reported that they thought some clients had carried on attending groups after they had ended contact, which they felt indicated that they had made an impact:

*“I went up there and physically supported them by standing next to them, and I also passed on the information on paper, and as far as I know they went on and carried it forward themselves.”* SW4.

Sometimes improvements were reported by SWs to be subtler, reflecting patients’ accounts the importance of feeling heard:

*“You know, I was just going along and sitting down and saying, ‘tell me, tell me, you know, I’ve got time and I want to listen and you can be as honest with me as you want, just tell me because I care about what you’re going to say’. And I think that is such a simple thing, but it is absolutely key, because just the fact that somebody would want to do that and be genuinely interested, even if you can’t help them resolve it or maybe only a bit, because with that participant we did make some progress.”* SW4.

The SWs suggested one of the main factors contributing to helping clients was the time they invested in people:

*“I think a lot of what was positive about this was, I think, the person visiting, just keeps showing that person that they are important and you do care about them and I think that was quite a motivating factor.”* SW3.

#### Reflecting on supervision

SWs reported that the one-to-one supervision offered was helpful in dealing with their anxieties, particularly after the first visit to the first client:

*“The first supervision that I did on the phone was very, reassuring…… just to, just to know that there was somebody else there who I could offload to myself, and that was welcomed.”* SW4.

*“[Supervision was] quite good really. They were all over the phone. I did think, I don’t know whether it was, we were supposed to have some supervision meetings. I initially thought that it would be a supervision session [right] somewhere. I didn’t think it would just be on the phone. But it was ok, you know, it was fine. But because I didn’t have any really major problems, it was just really talking about each case so, that was very useful, I think.”* SW5.

Face-to-face meetings were held between the SWs and CCG and HB, but these were seen more as group support meetings rather than as more formal ‘supervision’ sessions.

*What could be done differently?*

Some SWs felt that the intervention could be improved by building in a follow-up session with clients a few months after the six sessions:

*“Hopefully that there would be an opportunity to engage over a longer time, I think also, some kind of follow up could be built in, say at possibly a three month or six month point, perhaps a year or something like that.”* SW6.

Other SWs felt that the limited time of the intervention helped to motivate patients to make the most of the sessions:

*“More than one person said to me, well, I’ve only got you for a short time, so I better make good use of you, sort of thing, so, it spurred them in a way, really, they’re all, oh I’ve only got like four or five sessions, or six sessions so, if I’m going to do something, I’ve got to do it now type of thing , without making them feel rushed in anyway, but, it took, sometimes took a few weeks to get to that stage but, no, I thought it was really good.”* SW5.

Thus, SWs recognized that people in the study had complex problems, and to meet this challenge required the support of their peers on the study, colleagues at AgeUK and the opportunity to discuss at supervision. The knowledge and expertise that the SWs already had through previous training, and concurrent experience working within AgeUK, were perceived as vital in suggesting local groups to study participants, being able to contact the group leaders and feeling comfortable accompanying people to groups.

### Perspectives of GPs

The interviews with GPs identified themes around working with patients with complex needs, and GPs’ views on the study.

#### Older adults as a vulnerable group

GPs recognised that there is a group of depressed and/or lonely older people who may benefit from simply having a conversation with someone:

*“We used to have a receptionist here who was very astute, who would say ‘do you realise, if I had a kettle, teapot and a packet of biscuits, half these people coming in to see you wouldn’t need to come in and see you.”* GP practice 3.

*“Initially I think more face to face chat and probably seeing them every week and just giving them a bit of support and listening to them and acknowledging what’s happening.”* GP practice 1.

They reported, however, that they were unable to provide time to listen due to time constraints in time-limited consultations, and lack of capacity within the practice:

*“I think all she came to do was talk to me and that’s fine, and might have been fine in general practice 20 years ago, 25 years ago. It ain’t any more. It’s too task orientated, it’s too problem orientated and there’s too much pressure as the hospital dump more and more long term conditions on us to look after. We’ve just not got that… We’ve not got that capacity, we’ve not got that sort of pastoral effect we used to have. We’ve just not got time.”* GP practice 2.

GPs reported that they often had difficulty diagnosing mental health problems in this group of patients with complex needs, which they saw as different to working with younger populations:

*“We don’t diagnose that well because they have other conditions, like dementia…so we tend to forget about and it’s not probably screened. And also they don’t ask for support as much as the younger people do, and because older people actually they say ‘it’s just anxiety I’ll get over it’. Whereas younger people, they work, and their work stress and everything, they want to take some time off or they want a sick note, that sort of thing,”* GP practice 4.

#### Lack of services

Provision for GPs to refer depressed or lonely older people was very variable, with some GPs reporting good wellbeing services or partnerships with organisations such as AgeUK providing support, whilst others reported a lack of appropriate services that could be accessed in a timely manner:

*“I think the other thing we must consider is to find underlying depression and anxiety in elderly patients without making sure we’ve got adequate resources, to cope with this. It will be a shame to diagnose depression in an elderly patient and then it would be a further shame that then there’s nothing to help them and possibly that will make them worse really.”* GP practice 5.

Thus, whilst GPs recognized that older people were a vulnerable group, they suggested that they had little time to offer pastoral support to older people, and that there were limited resources to which to refer people to.

#### Engagement with the study

Some GPs were supportive of the concept behind the NOTEPAD study, but others did not like the idea of using ‘non-medical personnel’ to work with patients with mental health problems, especially when the responsibility for suicide risk remained with the GP. This was felt particularly strongly in the context of cuts to secondary care psychiatric services in the local area [this was documented in field notes, summarising a conversation recorded prior to an interview].

Some GPs reported feeling annoyed by the NOTEPAD risk protocol which required a member of the research team to notify them if one of their patients expressed suicidal ideation. Some GPs felt that risk was being raised unnecessarily:

*GP: I had contact with the [researcher] who was worried that the patient had suicidal thoughts. I think I’ve had two or three of those and the patient had to speak to me but that was just depressing thoughts they were having. The [researcher] was very worried but the patient wasn’t.*

*I:* Did that annoy you at all?

*GP: Yeah, a bit…..a lot, actually.* GP practice 2.

Other than being contacted by researchers as part of the risk protocol, GPs did not feel that participating in the study had impacted on their routine practice. They recalled no contact with the SWs, and did not recollect any patients discussing participation in the study with them.

## Discussion

### Summary of results

This study is, to our knowledge, the first study which attempted to train SWs from AgeUK to deliver a psychosocial intervention to older people with anxiety and/or depression, recruited from primary care.

Analysis of digital recordings of SW-study participant sessions suggested that the SWs could be trained to deliver the intervention as intended.

Older people recruited to the study disclosed long-standing mental health problems together with physical health problems. This complexity was echoed by the SWs and GPs interviewed. The SWs described additional challenges posed by social situations that could include caring responsibilities, family stresses and financial worries. Despite this complexity, older people reported that the SW intervention was useful in a variety of ways, emphasising the value of being listened to and having someone interested in them, and spending time with them.

That the SW visited the older person at home was felt to be valuable by both older people and the SWs. The older person also valued information given by the SWs and in the resources provided. Some older people found sign-posting to groups, with the offer of accompanying to a group, acceptable, particularly when this group was felt to be personally meaningful. Other study participants did not feel that a group was suitable or appropriate for them, either because they were not interested in attending groups where they felt they were ‘passive recipients’ of a service, or because they did not feel that loneliness or social isolation were problems for them. The flexibility of the SW intervention allowed negotiation by the SWs with their clients, about attending a group. This person-centredness was valued by the older people interviewed and the SWs. All participants described positive experiences of meeting with the SWs; they described and valued the positive personal qualities of their individual SW, and appreciated the opportunity to talk to, and be listened to by, the SW. Such support was not seen to be available either within their family, or from statutory services. The GP was not seen as potential source of support.

Analysis of the data generated from interviews with SWs suggested that the training was acceptable to the SWs, who valued the SW manual, however after training and prior to first contact with participants the SWs experienced some apprehension. The SWs reported that the intervention offered older people the opportunity to talk, and that this could be seen to legitimize their problems and concerns. Giving time and empathy was seen to ensure that older people developed trust with the SW. Whilst the SWs reported that the prospect of assessing risk of self-harm and suicide had made them feel uncomfortable during the training, they reported that they had developed confidence when doing this in practice. The knowledge and expertise that the SWs already had through previous training, and concurrent experience working within AgeUK, were perceived as vital in suggesting local groups to study participants, being able to contact the group leaders and feeling comfortable accompanying people to groups. The SWs described the positive feelings they themselves experienced when they felt they had made a difference to the study participants.

The GPs interviewed reported that they had little understanding of the study and were not aware of what the intervention entailed or the content of the interaction between SWs with their patients. Practice participation in the study had not impacted on their routine work, apart from the need to respond to the research team when participants expressed suicide ideation.

### Comparison with previous literature

Pettit et al. [47] report that older adults are still under-represented in IAPT services, and the NICE guideline 123 [27] suggests the need to modify interventions to improve access. It is important to establish whether changes to service configuration, treatment options, and GP behaviour can increase referrals for middle-aged and older adults. In response to the knowledge that older people are a vulnerable group for whom access to MH services needs to improve, the AMP research programme [38] developed a model to improve access and develop acceptable interventions. In the AMP study [39], one of the vulnerable groups in which a new model of care was evaluated was older people with depression, but the intervention was delivered by IAPT practitioners who worked closely with local groups offered by Age Concern. This study was an attempt to increase access to care for people with anxiety and depression by developing and testing the acceptability of less stigmatising intervention delivered by SWs from the third sector.

In the CASPERplus trial [48], it was reported that offering older people an opportunity to talk outside the primary care consultation was valued by patients and GPs and that psychosocial intervention in the broader primary care setting may fill the gap in the care of older people with depression. Our findings confirm this. Similarly, evaluation of a service development in which Practice Nurses deliver a psychosocial intervention to patients with long-term conditions, suggests that patients valued the time and availability of Practice Nurses to listen to their concerns [49].

Patients with long-term conditions and co-morbid depression in the COINCIDE trial [50], preferred a protected space to discuss mental health issues, and study participants in this study alluded to their beliefs that they did not feel it was appropriate to discuss low mood or stress and distress with their GP. The Practice Nurses and IAPT practitioners in the COINCIDE trial expressed a wish to maintain barriers around physical and mental health expertise. In our study, GPs suggested that they could not offer support to older people with anxiety and depression, and lacked services to refer them to. The SW role might meet this need.

The GPs interviewed as part of the process evaluation suggested that the study had only impacted on the practice when a call was received in relation to the risk protocol. The SWs had no contact with the practices from which study participants were recruited. Whilst a safe space perceived to be outside the primary care consultation was valued, it might have been better if we had achieved closer liaison between the SW and practices, perhaps linking one SW with one practice. This was achieved in the ‘Deep End’ scheme [51] in Glasgow where ‘community links practitioners’ (CLPs) were embedded in practices; these CLPs carried out which one-to-one working with patients to support patients’ use of community services.

This study has improved our understanding of loneliness, people’s response to it, and the potential role of the third sector. The qualitative study by Kharicha and colleagues [32] suggested that older people with characteristics of loneliness generally know about local resources but may not consider services they perceive as being for ‘lonely older people’, as desirable, helpful or relevant to them. Our study suggests that groups can be acceptable to some people who perceive themselves to be lonely: group-based activities with a shared interest and purpose are preferred. Our study resonates with Kharichi’s findings that older people experiencing loneliness may not consider that primary care has a role in alleviating this.

### Strengths and limitations

A strength of this study is the PPIE input throughout the study, with input into the original funding application, comments on the proposed intervention, drafting public-facing documents, Support Worker training and manual, patient resources, advice on documents for ethics applications, reflections on analysis and advice on dissemination.

In terms of limitations, it should be noted that the fidelity checks were not completed by individuals independent to the feasibility study. A limitation of the process evaluation is the difficulty we encountered recruiting GPs to the interview, which resulted in often short interviews, sometimes over the telephone and sometimes in groups.

## Conclusions

Support Workers, recruited from AgeUK, North Staffordshire, were capable of working with older adults with anxiety and depression and delivering the psychosocial intervention as intended. The intervention was acceptable to older adults; the personal qualities of the SWs were valued; the intervention was perceived to be less stigmatising than statutory services. Sign-posting to group activities was not acceptable to all older adults; and older adults did not want to be passive recipients of services, preferring a more reciprocal relationship.

It is important that the expertise that already exists in third sector service staff is recognised and utilised within primary care. SWs have the potential to deliver a non-stigmatising, low-level psychosocial intervention to support and manage older people with anxiety and depression, potentially useful within a resource-poor NHS.

## Additional files

Additional file 1: Fidelity checklist patient participants. (DOCX 16 kb)

Additional file 2: Topic guide for patient participants in intervention arm. (DOCX 14 kb)

Additional file 3: Topic guide for patient participants who dropped out of study. (DOCX 14 kb)

Additional file 4: Topic guide for GP participants. (DOCX 14 kb)

Additional file 5: Topic guide for SW participants. (DOCX 14 kb)

## Abbreviations

AMP: Access to Mental Health in Primary Care
BA: Behavioural Activation
CASPER: CollAborative care and active surveillance for Screen-Positive EldeRs with subthreshold depression
COINCIDE: Collaborative Interventions for Circulation and Depression
GP: General Practitioner
IAPT: Improving Access to Psychological Therapies
NHS: National Health Service
NICE: National Institute for Health and Care Excellence
NIHR: National Institute for Health Research
NOTEPAD: NOn-Traditional providers to support the management of Elderly People with Anxiety and Depression
PIP: Personal Independence Payments
PPIE: Patient and Public Involvement and Engagement
RN: Research Nurse
SW: Support Worker
