# Supplementary material for: Can support workers from AgeUK deliver an intervention to support older people with anxiety and depression? A qualitative evaluation
Source: BMC Fam Pract. 2019 Jan 19;20:16. doi: 10.1186/s12875-019-0903-1 (PMC6339431; doi:10.1186/s12875-019-0903-1)
Supplement: Supplementary file 1 — Fidelity checklist patient participants. (DOCX 16 kb) [file 12875_2019_903_MOESM1_ESM.docx]

| **NOTEPAD Fidelity Checklist –** First session | YES | Partially | NO | | N/A |
| --- | --- | --- | --- | --- | --- |
| Verbal explanation given of the NOTEPAD study |  |  |  | |  |
| Explanation of the evidence for the beneficial effects of social participation and depression |  |  |  | |  |
| Evidence of exploring the older person’s problems |  |  |  | |  |
| Assessment of risk |  |  |  | |  |
| Activities/social participation goals discussed |  |  |  | |  |
| Activity/social participation goals set |  |  |  | |  |
| The NOTEPAD personal file given along with a verbal explanation of how to use it |  |  |  | |  |
| Signposting – (e.g. exercise groups, craft classes etc.) |  |  |  | |  |
| Participant understanding of what has been discussed and agreed is checked |  |  |  | |  |
| Barriers/motivators to increasing activity discussed and/or addressed |  |  |  |  | |
| Next session discussed and arranged (face to face or telephone) |  |  |  |  | |

| **NOTEPAD Fidelity Checklist –** Second sessions | YES | Partially | NO |
| --- | --- | --- | --- |
| Review mood - mood thermometers |  |  |  |
| Review progress - diary |  |  |  |
| Feedback given regarding any progress made |  |  |  |
| Barriers/motivators to increased activity / participation discussed and/or addressed |  |  |  |
| Activity/social participation goals discussed |  |  |  |
| Activity/social participation goals set |  |  |  |
| Signposting – (e.g. craft groups, adult learner classes etc.) |  |  |  |
| Remind about use of NOTEPAD personal file |  |  |  |
| Relapse prevention / staying well strategies discussed (e.g. support and guidance) |  |  |  |
| Possible personal issues/difficulties encountered whilst increasing activity/social participation. |  |  |  |
| Relevant contact details are given in case of any problems, issues or further advice required |  |  |  |

| **NOTEPAD Fidelity Checklist –** Subsequent sessions | YES | Partially | NO |
| --- | --- | --- | --- |
| Review mood – mood thermometers |  |  |  |
| Review progress (diary) |  |  |  |
| Feedback given regarding any progress made |  |  |  |
| Barriers/motivators to increased activity / participation discussed and/or addressed |  |  |  |
| Activity/social participation goals discussed |  |  |  |
| Activity/social participation goals set |  |  |  |
| Signposting – (e.g. craft groups, adult learner classes etc.) |  |  |  |
| Remind about use of NOTEPAD personal file |  |  |  |
| Relapse prevention / staying well strategies discussed (e.g. support and guidance) |  |  |  |
| Possible personal issues/difficulties encountered whilst increasing activity/social participation. |  |  |  |
| Relevant contact details are given in case of any problems, issues or further advice required |  |  |  |
