# Supplementary material for: Can support workers from AgeUK deliver an intervention to support older people with anxiety and depression? A qualitative evaluation
Source: BMC Fam Pract. 2019 Jan 19;20:16. doi: 10.1186/s12875-019-0903-1 (PMC6339431; doi:10.1186/s12875-019-0903-1)
Supplement: Supplementary file 2 — Topic guide for patient participants in intervention arm. (DOCX 14 kb) [file 12875_2019_903_MOESM2_ESM.docx]

- Their overall perspectives of the intervention
- Whether the questionnaires were acceptable
- Participant equipoise and propensity to undergo randomisation in a pilot RCT
- How acceptable and useful participants found the one-to-one sessions with the SW
- Preference of face to face or telephone intervention sessions
- What participants recalled doing with the SW
- Whether they attended any groups, and whether (and how) this was facilitated by the SW
- How closely the group interventions matched the interests of the participants
- How acceptable and useful participants found the group sessions
- Satisfaction with the group intervention
- If the intervention was flexible enough to meet their needs
- Whether participants had previous contact with services and reasons why they did or did not
- Barriers and facilitators to their (non-)engagement with the SW or with groups
- Use of participant manual (NOTEPAD file) and diaries
- Whether their engagement in a group has continued
