# Supplementary material for: Can support workers from AgeUK deliver an intervention to support older people with anxiety and depression? A qualitative evaluation
Source: BMC Fam Pract. 2019 Jan 19;20:16. doi: 10.1186/s12875-019-0903-1 (PMC6339431; doi:10.1186/s12875-019-0903-1)
Supplement: Supplementary file 3 — Topic guide for patient participants who dropped out of study. (DOCX 14 kb) [file 12875_2019_903_MOESM3_ESM.docx]

- Reasons for interest in study
- Whether the questionnaires were acceptable
- Participant equipoise and propensity to undergo randomisation in a pilot RCT
- Reasons for discontinuing
- Overall perspectives of the intervention
- Preference of face to face or telephone intervention sessions
- How acceptable and useful participants found the one-to-one sessions with the SW
- What participants recalled doing with the SW
