# Supplementary material for: Can support workers from AgeUK deliver an intervention to support older people with anxiety and depression? A qualitative evaluation
Source: BMC Fam Pract. 2019 Jan 19;20:16. doi: 10.1186/s12875-019-0903-1 (PMC6339431; doi:10.1186/s12875-019-0903-1)
Supplement: Supplementary file 4 — Topic guide for GP participants. (DOCX 14 kb) [file 12875_2019_903_MOESM4_ESM.docx]

- Their perspectives on managing older people with anxiety and depression
- Experiences, barriers and facilitators to working with the third sector
- Clinician’s position of equipoise regarding the treatment arms
- Any feedback they have received from older patients involved in NOTEPAD
- Any contact with the SWs or study team, and impact on themselves and the practice
- How/if their management of older people with anxiety and/or depression has changed
