# Supplementary material for: Can support workers from AgeUK deliver an intervention to support older people with anxiety and depression? A qualitative evaluation
Source: BMC Fam Pract. 2019 Jan 19;20:16. doi: 10.1186/s12875-019-0903-1 (PMC6339431; doi:10.1186/s12875-019-0903-1)
Supplement: Supplementary file 5 — Topic guide for SW participants. (DOCX 14 kb) [file 12875_2019_903_MOESM5_ESM.docx]

- Their reflections on NOTEPAD – the intervention, working with research team
- Their experiences of training
- Their experience of delivering the NOTEPAD intervention to older people; their attitudes to outcome assessment; experience of assessing and dealing with risk; sign-posting to groups
- Views on the SW manual and client resources, NOTEPAD file, use of diaries, use of mood thermometers
- Their experiences of support and supervision
- Their views on liaison with primary care (if, this took place) and other statutory agencies (e.g. IAPT)
- Barriers and facilitators to implementation of such an intervention
